# Selection of Reference Genes for MicroRNA Quantitative Expression Analysis in Chinese Perch, *Siniperca chuatsi*

**DOI:** 10.3390/ijms16048310

**Published:** 2015-04-14

**Authors:** Xin Zhu, Yu-Long Li, Dun-Xue Chen, Ping Wu, Tan Yi, Tao Chen, Jian-She Zhang, Wu-Ying Chu

**Affiliations:** 1 Department of Bioengineering and Environmental Science, Changsha University, Changsha 410003, China; E-Mails: xinzhu1219@163.com (X.Z.); liyulong0801@163.com (Y.-L.L.); chendunxue@163.com (D.-X.C.); wuping055@163.com (P.W.); yitan724@126.com (T.Y.); 2 College of Veterinary Medicine, Hunan Agriculture University, Changsha 410128, China; E-Mail: chentao@hunau.edu.cn; 3 Collaborative Innovation Center for Efficient and Health Production of Fisheries in Hunan Province, Changde 415000, China

**Keywords:** miRNA, real-time PCR, reference genes, relative quantification, *Siniperca chuatsi*

## Abstract

Real-time quantitative reverse transcription PCR (RT-qPCR) is one of the most effective and sensitive techniques in gene expression assay, for which selection of reference genes is a prerequisite. In teleost species, such as Chinese perch, the expression profiling of miRNAs as reference genes for RT-qPCR has not been intensively studied. In the present study, the expression profiles of six miRNAs (miR-101a, miR-146a, miR-22a, miR-23a, miR-26a and let-7a) and one small nuclear RNA (U6) were assayed with RT-qPCR in different adult tissues, developmental stages and growth conditions of Chinese perch, *Siniperca chuatsi*. The analyses revealed that embryonic developmental stage is an important variability factor in the expression stability of miRNAs. All six miRNAs exhibited better expression consistency than U6 in most of the conditions examined, and therefore, they may be more suitable as a reference gene for miRNA quantification. When different tissues and developmental stages were considered, miR-22a demonstrated the most consistent expression pattern, and the best combination of reference genes was miR-22a and miR-23a. Our study offers useful data for selecting miRNAs as reference genes for RT-qPCR analysis of miRNAs in teleost fishes under different conditions.

## 1. Introduction

MicroRNAs (miRNAs) are approximately 22-nt non-coding RNAs and they act as negative regulators of gene expression either by inhibiting mRNA translation or promoting mRNA degradation through partial base pairing at the 3'-untranslated region (3'-UTR) of the target mRNAs [1,2]. miRNAs play a key role in diverse biological processes such as organ development, cell proliferation, tumorigenesis, fat metabolism, behavior and embryogenesis [3,4]. Furthermore, abnormal expression of miRNAs may result in disease, dramatic phenotype variation, and even death [5]. It is apparent that assays of expression patterns of miRNAs in development, disease and other cellular processes exhibit both theoretical and practical significances. However, the crucial issue for the assay is to select suitable reference genes for accurate calibration.

Several modern technologies, such as microarrays, high-throughput sequencing, bead-based flow-cytometry, northern blot and RT-qPCR have been used for determination and quantification of miRNA expression [6,7,8]. The advantages of RT-qPCR for gene expression analysis are sensitivity and relatively accurate, wide dynamic range, relatively low-cost and low template requirements [9]. Different strategies have been used to normalize RT-qPCR data [10,11,12], and selection of reliable reference genes as internal controls has to be carefully considered before any experimental design [13]. However, certain criteria have to be fulfilled for reference gene selection [12,14]. The stability of a given reference gene should be experimentally validated in each study. miRNA data normalization has usually relied on some other small RNA expression analyses, such as small nuclear (snRNAs) or small nucleolus (snoRNAs) RNAs (e.g., U8, U6 or RNU44). However, there are some potential disadvantages in stability for these references as they are produced and/or processed by different pathways [12]. Recently, a few studies have explored the stability of some miRNAs in vertebrate species. Davoren [9] reported that let-7a and miR-16 were reliable for gene expression normalization in human breast cancer studies. Let-7a, miR-17-5p, miR-103 and miR-26 were confirmed as the most stable miRNA in different specific tissues of rat [15,16]. In grass carp, miR-101a was showed as the most stable in all tissues and different developmental stages among the other miRNAs they studied [17].

The Chinese perch (*Siniperca chuatsi*) is one of the most commercially important carnivorous fish species in China as well as in eastern Asia [18,19]. In the study, the expression profile of six candidate reference miRNA genes (miR-101a, miR-146a, miR-22a, miR-23a, miR-26a and let-7a) were examined in 14 developmental stages, 8 tissues and the liver tissues from fasting-refeeding experiment of Chinese perch. U6 belongs to small nuclear RNA, and is commonly used as reference gene in miRNA quantification [20]. A comparison assay of U6 with our candidate reference miRNAs was also conducted. The consistency of the best-scoring reference miRNA was assayed by the four statistical approaches (geNorm, BestKeeper, comparative Δ-*C*_t_ method and NormFinder). The results revealed that all of the six miRNAs exhibited valid stability values and could be used as reference miRNAs in some of the tested conditions, and they were more suitable as reference gene for miRNA quantification than U6 in most of the tested conditions. Therefore, our study offers a comprehensive value of mRNA reference selection for RNAs expression assays at different conditions in teleost fish species.

## 2. Results

### 2.1. RT-qPCR Assay Validations

miR-101a, miR-146a, miR-22a, miR-23a, miR-26a, let-7a and U6 were selected to assay their expression stability in different adult tissues, different embryonic and post-embryonic developmental stages and the fasting-refeeding treated liver tissues. All candidate reference miRNAs were successfully amplified with RT-qPCR with efficiencies ranging from 96.5%–103.1%. Thus, all of the miRNAs and U6 were considered to be acceptable for the next assay (Table 1).

**Table 1:** Sequence and primer information for seven selected candidate reference genes.

Name	Sequence (5'→3')	Primer Sequence (5'→3')	Amplification Efficiency
miR-22a-F	AAGCUGCCAGCUGAAGAACUGU	AGCTGCCAGCTGAGACTGT	97.5%
miR-23a-F	AUCACAUUGCCAGGGAUUUCCA	CATCACATTGCCAGGGATTTC	98.1%
miR-26a-F	UUCAAGUAAUCCAGGAUAGGCU	CGTTCAGTATCCAGGATAGGCT	97.8%
miR-146a-F	UGAGAACUGAAUUCCAUAGAUGG	CGTGAGACTGATTCCATAGATGG	96.5%
miR-101a-F	UACAGUACUGUGAUAACUGAAG	CGGTACAGTACTGTGATAACTGAAG	103.1%
let-7a-F	UGAGGUAGUAGGUUGUAUAGUU	CGGTGAGGTAGTAGGTTGTATAGTT	102.3%
U6-Forward	–	CTCGCTTCGGCAGCACA	99.3%
U6-Reverse	–	AACGCTTCACGAATTTGCGT	–

Forward primers used for detection were detailed in the Methods; The reverse primer used for miRNA detection was universal downstream primer (Uni-miR qPCR Primer, 10 μmoL/L, Takara).

### 2.2. Expression Levels of Reference miRNAs under Different Conditions

**Figure 1:**
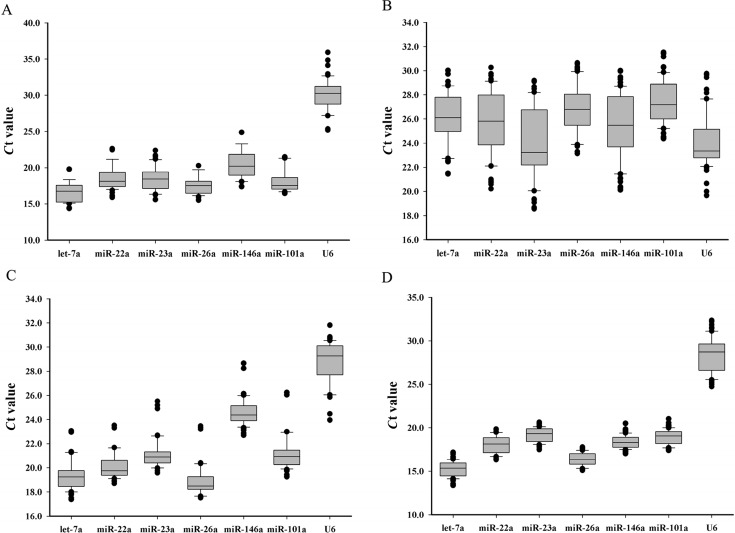
Threshold cycles (*C*_t_) comparison of six reference miRNAs and U6. (**A**) In different adult tissues; (**B**) In different embryonic developmental stages; (**C**) In different post-embryonic developmental stages; and (**D**) In fasting-refeeding treated liver tissue.

RT-qPCR was performed to evaluate the expression patterns of the candidate reference miRNAs and U6 in different adult Chinese perch tissues, different developmental stages and the fasting-refeeding treated liver tissues of Chinese perch. The same amount of total RNA from the different embryo and tissue samples was reverse transcribed in triplicates. The obtained data showed that the expression level of U6 is much lower than any of the six miRNAs in most instances, but had some variation in different embryonic developmental stages (Figure 1). The expression levels of six miRNAs and U6 displayed a wide range of cycle threshold (*C*_t_) values ranging from 14.35 to 35.87 in different adult tissues (Figure 1A). Among the six miRNAs assayed, let-7a was the most highly expressed with the lowest *C*_t_ value at 14.35 (Figure 1A) and miR-26a showed the least variability, with an expression variation range at 4.78 cycles (Figure 1A). For the different embryonic stages assayed, the expression of the U6 and six reference miRNAs showed *C*_t_ values ranging from 18.54 to 31.53 (Figure 1B). Among of them, miR-23a was the highest expressed with the lowest *C*_t_ value at 18.54, while miR-101a showed the least variability with an expression variation range at 7.05 cycles (Figure 1B). Meanwhile, the six miRNAs and U6 expression showed *C*_t_ values ranging from 17.38 to 30.84 in fast muscles of different post-embryonic developmental stages (Figure 1C). Of them, let-7a was the most highly expressed with the lowest *C*_t_ value at 17.36, while miR-26a showed the least variability with a variation range at 5.03 cycles (Figure 1C). Furthermore, the six miRNAs expression showed *C*_t_ values ranging from 13.37 to 32.34 in the fasting-refeeding treated liver tissue (Figure 1D). While the maximal of six miRNAs expression variation range during fasting-refeeding experiment was 3.79, which was less than the minimum expression variation ranges (minimum variation range was 4.78) compared to those of either different tissues or developmental stages.

### 2.3. Analysis of the Stability of the Reference miRNAs

**Figure 2:**
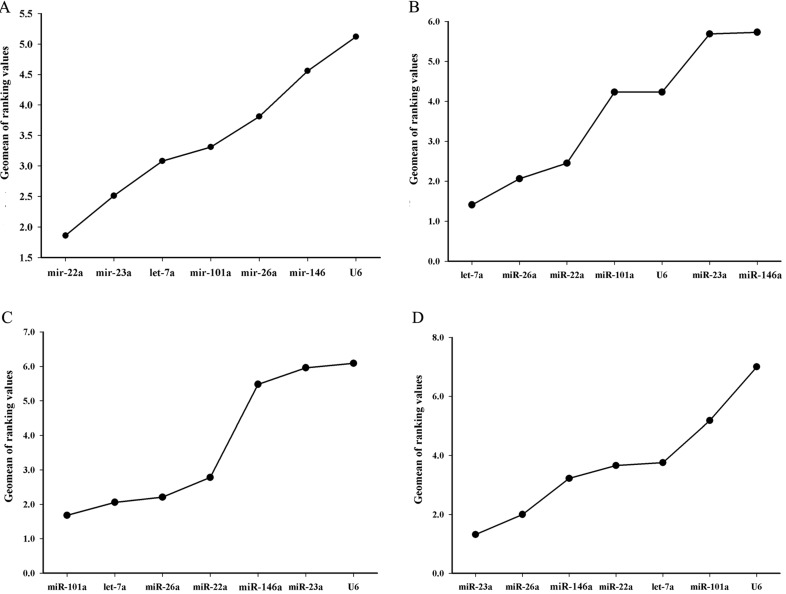

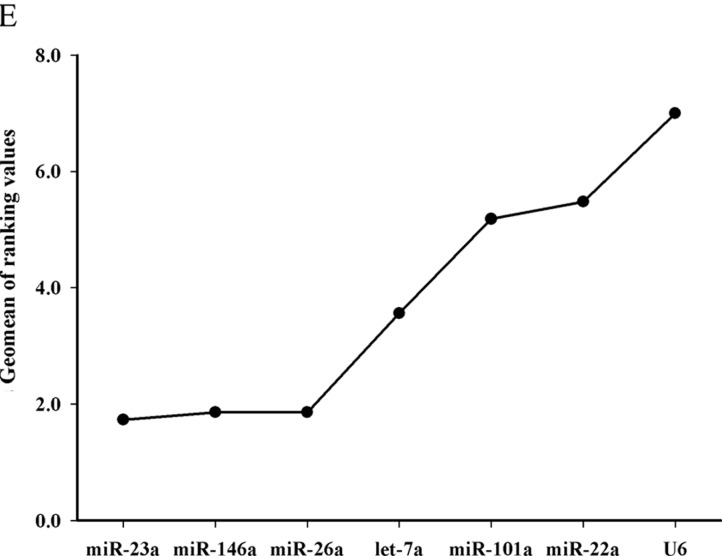
The recommended comprehensive ranking values calculated by RefFinder. (**A**) The stability was evaluated based on the entire data (all tissues, embryonic and post-embryonic developmental stages and fasting-refeeding experiment); (**B**) Different tissues. (**C**) Different embryonic developmental stages; (**D**) Different post-embryonic developmental stages; and (**E**) Fasting-refeeding treated.

To find the best reference genes with the highest stability and the lowest biological variance for an accurate gene expression analyses, it is necessary to assay their stability. For this purpose, the most frequently used programs including BestKeeper, geNorm, NormFinder and the comparative Δ-*C*_t_ method were applied. The detailed stability values obtained for each miRNA by BestKeeper, NormFinder and the comparative Δ-*C*_t_ algorithm in consideration of all samples are shown in Part 1, Tables S1–1 to S1–4 (Supplemental Material); The detailed stability values for each miRNA by three algorithm in consideration of different adult tissues are shown in Part 2, Tables S2–1 to S2–4 (Supplemental Material); The detailed stability values for each miRNA by three algorithm in consideration of different embryonic developmental stages are shown in Part 3, Tables S3–1 to S3–4 (Supplemental Material); The detailed stability values for each miRNA by three algorithm in consideration of different post-embryonic developmental stages are shown in Part 4, Tables S4–1 to S4–4 (Supplemental Material); The detailed stability values for each miRNA by three algorithm in consideration of fasting and refeeding experiment are shown in Part 5, Tables S5–1 to S5–4 (Supplemental Material). As shown in Table 2 and Figure 2A, let-7a was the most stably expressed in the different tissues, followed by miR-26a, miR-22a, miR-101a, U6, miR-23a and miR-146a in sequencing (Figure 2B). The *M*-value of the miRNAs in different tissues calculated by geNorm showed a good stability value ranging from 0.708 to 1.447 (*M*-values < 1.5), indicating that these miRNAs were suitable reference miRNAs for different tissues (Table 3). But the *M*-value of U6 in different tissues showed a poor stability value with 2.23. However, for those in the different embryonic developmental stages, the order of gene expression stability from the high to low was miR-101a, let-7a, miR-26a, miR-22a, miR-146a, miR-23a and U6 (Figure 2C) in sequencing (Table 4). In different post-embryonic developmental stages, the comprehensive ranking from high to low was miR-23a, miR-26a, miR-146a, miR-22a, miR-101a, let-7a and U6 (Figure 2D). For the fasting and refeeding experiment, the variation of six candidate reference miRNAs was ranged as miR-23a, miR-146a, miR-26a, let-7a, miR-101a, miR-22a to U6 (Figure 2E), and the stability values of the six miRNAs were less than 1.5 (Table 6). The results of comparative profiling of the miR-499 expression in white and red muscle by microarray analysis and RT-qPCR are shown in Table 7. When miR-22a was used as a reference gene, the relative quantitative result was similar to the microarray analysis result.

Table 2Ranking of candidate reference miRNAs and U6 in consideration of all samples including tissues, developmental stages and fasting and refeeding experiment.

**Table:** **A**

Method	Ranking Order (Better-Good-Average)						
	1	2	3	4	5	6	7
Δ-*C*_t_	miR-22a	miR-101a	let-7a	miR-23a	miR-26a	miR-146a	U6
BestKeeper	miR-23a	U6	miR-22a	miR-146a	miR-101a	let-7a	miR-26a
Normfinder	miR-22a	miR-23a	miR-146a	miR-101a	let-7a	miR-26a	U6
geNorm	let-7a/miR-26a	–	miR-101a	miR-22a	miR-23a	miR-146a	U6

**Table:** **B**

Gene Name	let-7a/miR-26a	miR-101a	miR-22a	miR-23a	miR-146a	U6
geNorm Stability value	0.981	1.216	1.334	1.683	1.874	3.114

Ranking of candidate reference genes according to stability values produced by geNorm, NormFinder, BestKeeper and Δ-*C*_t_, and the recommended comprehensive ranking calculated by RefFinder. **A**: Rank order of each miRNA in consideration of all samples including tissues, developmental stages and fasting and refeeding experiment; and **B**: The geNorm stability value of each miRNA in consideration of all tissues and developmental stages.

Table 3Ranking of candidate reference miRNAs and U6 in different adult tissues.

**Table:** **A**

Method	Ranking Order (Better-Good-Average)						
	1	2	3	4	5	6	7
Δ-*C*_t_	let-7a	miR-26a	miR-22a	miR-101a	miR-23a	miR-146a	U6
BestKeeper	U6	miR-22a	miR-26a	let-7a	miR-101a	miR-146a	miR-23a
Normfinder	let-7a	miR-22a	miR-26a	miR-101a	miR-146a	miR-23a	U6
geNorm	let-7a/miR-26a	–	miR-22a	miR-101a	miR-23a	miR-146a	U6

**Table:** **B**

geNorm	let-7a/miR-26a	miR-22a	miR-101a	miR-23a	miR-146a	U6
geNorm Stability value	0.708	0.867	1.086	1.291	1.447	2.230

Ranking of candidate reference genes according to stability values produced by geNorm, NormFinder, BestKeeper and Δ-*C*_t_,and the recommended comprehensive ranking calculated by RefFinder. **A**: Rank order of each miRNA in different tissues; and **B**: The geNorm stability value of each miRNA in different tissues.

Table 4Ranking of candidate reference miRNAs and U6 in different embryonic developmental stages.

**Table:** **A**

Method	Ranking Order (Better-Good-Average)						
	1	2	3	4	5	6	7
Δ-*C*_t_	miR-22a	let-7a	miR-26a	miR-101a	miR-146a	miR-23a	U6
BestKeeper	miR-101a	miR-26a	let-7a	U6	miR-22a	miR-146a	miR-23a
Normfinder	let-7a	miR-101a	miR-22a	miR-26a	miR-23a	miR-146a	U6
geNorm	miR-26a/miR-101a	–	let-7a	miR-22a	miR-146a	miR-23a	U6

**Table:** **B**

Gene Name	miR-26a/miR-101a	let-7a	miR-22a	miR-146a	miR-23a	U6
geNorm Stability value	0.804	0.824	0.875	0.934	0.985	1.369

Ranking of candidate reference genes according to stability values produced by geNorm, NormFinder, BestKeeper and Δ-*C*_t_,and the recommended comprehensive ranking calculated by RefFinder. **A**: Rank order of each miRNA in embryonic development stages; and **B**: The geNorm stability value of each miRNA in embryonic development stages.

Table 5Ranking of candidate reference miRNAs and U6 in different post-embryonic developmental stages.

**Table:** **A**

Method	Ranking Order (Better-Good-Average)						
	1	2	3	4	5	6	7
Δ-*C*_t_	miR-23a	miR-26a	miR-22a	let-7a	miR-101a	miR-146a	U6
BestKeeper	miR-146a	let-7a	miR-23a	miR-26a	miR-22a	miR-101a	U6
Normfinder	miR-23a	miR-26a	miR-22a	miR-101a	let-7a	miR-146a	U6
geNorm	miR-23a/miR-26a	–	miR-146a	miR-22a	let-7a	miR-101a	U6

**Table:** **B**

Gene Name	miR-23a/miR-26a	miR-22a	miR-101a	let-7a	miR-146a	U6
geNorm Stability value	0.546	0.623	0.661	0.707	0.742	1.375

Ranking of candidate reference genes according to stability values produced by geNorm, NormFinder, BestKeeper and Δ-*C*_t_, and the recommended comprehensive ranking calculated by RefFinder. **A**: Rank order of each miRNA in post-embryonic development stages; and **B**: The geNorm stability value of each miRNA in post-embryonic development stages.

Using a single reference gene for gene expression analysis may not meet the requirements. Therefore, we further examined two or more reference genes in this context. As assayed by NormFinder, the data indicated that the best combination of miR-22a and miR-23a for different tissues, miR-22a and miR-146a for different embryonic developmental stages, let-7a and miR-26a for post-embryonic developmental stages of muscle, and miR-26a and miR-23a for the fasting-refeeding treated liver tissue. The stability values of the above combinations were 0.320, 0.338, 0.191, 0.085 and 0.158, respectively.

Table 6Ranking of candidate reference miRNAs and U6 during fasting and refeeding experiment.

**Table:** **A**

Method	Ranking Order (Better-Good-Average)						
	1	2	3	4	5	6	7
Δ-*C*_t_	miR-23a	miR-26a	miR-146a	let-7a	miR-22a	miR-101a	U6
BestKeeper	miR-146a	miR-26a	miR-23a	miR-101a	let-7a	miR-22a	U6
Normfinder	miR-23a	let-7a	miR-26a	miR-146a	miR-22a	miR-101a	U6
geNorm	miR-146a/miR-26a	–	miR-23a	let-7a	miR-101a	miR-22a	U6

**Table:** **B**

geNorm	miR-146a/miR-26a	miR-23a	let-7a	miR-101a	miR-22a	U6
geNorm Stability value	0.456	0.527	0.560	0.729	0.856	1.220

Ranking of candidate reference genes according to stability values produced by geNorm, NormFinder, BestKeeper and Δ-*C*_t_,and the recommended comprehensive ranking calculated by RefFinder. **A**: Rank order of each miRNA during fasting and refeeding experiment; and **B**: The geNorm stability value of each miRNA during fasting and refeeding experiment.

**Table 7:** Comparative profiling of the miR-499 expression in white muscle and red muscle of *Siniperca chuatsi* by microarray analysis and RT-qPCR.

Method	White Muscle	Red Muscle	Relative(*R*/*W*)
Microarray Analysis	96	4290	44.69
Reference miRNA	–	–	–
U6	1	106.82	106.82
miR-22a	1	45.38	45.38
Let-7a	1	41.26	41.26
miR-26a	1	34.47	34.47
miR-101	1	17.93	17.93
miR-146a	1	14.53	14.53
miR-23a	1	3.28	3.28

## 3. Discussion

As reported in previous studies, the two most commonly used reference genes for miRNA RT-qPCR are U6 and 18S RNA [20,21,22]. Because rRNAs could be expressed at much greater levels than target RNAs, bias might exist for lower-expressed target RNA expression quantification [14,23]. The 18S rRNA has been used as reference gene, but it also showed some variations because of its high abundance [24]. Therefore, it is obvious that the same class of RNA should be selected for miRNA quantification. To date, study on the suitability of reference genes for miRNA expression in teleost is limited [17].

In the study, six miRNAs (miR-22a, miR-146a, miR-101a, let-7a, miR-23a and miR-26a) were selected to assay their suitability in different tissues, different developmental stages and the fasting-refeeding treated liver tissue. The six miRNA sequences were obtained from the Chinese perch miRNA library reported by Chu [25]. With the multiple analyses by geNorm, BestKeeper, Normfinder and Δ-*C*_t_, the data revealed that miR-22a, let-7a, miR-101a and miR-26a are the best reference miRNA genes based on their average expression stability (*M*) or stability values. The geNorm program was used to calculate the *M* stability value of a gene based on the average pairwise variation between all the studied genes. High gene expression variability results in high *M*-values and exhibits low expression stability. If the *M*-value of the evaluated genes is below 1.5, it indicates that the expression of the candidate gene is relatively stable [14,16,17]. Our data showed that the *M*-value of miR-23a, miR-146a and U6 were 1.683, 1.874 and 3.114 (*M*-values > 1.5), respectively, indicating that they are not suitable as reference miRNAs in all instances. We further ran a validation experiment with comparative profiling of the miR-499 expression in white and red muscle of *Siniperca chuatsi* by microarray analysis and RT-qPCR to validate our reference miRNAs. As evident from our results, inappropriate use of reference genes can significantly alter the relative expression of miR-499 between white and red muscle. When using miR-22a or let-7a as reference gene, the quantitative result was most similar to microarray analysis data between white and red muscle.

Tissue specificity may be an important factor that affects the stability of miRNA expression. In the present study, the stability of the miRNAs was evaluated for each of the eight tissues. By geNorm analysis, the stability values ranged from 0.708 to 1.447 across the different tissues (Table 3). The results suggested that all the candidate reference miRNAs could be used for tissue-specific expression assays, but the best reference miRNA was let-7a (Figure 2B), which was consistent to those of earlier reports. Especially, let-7a was confirmed to be a reliable reference gene in human breast and colorectal cancer [9,26]. Let-7a was also detected as the most stable miRNA in skeletal muscle and ovary of pig [16,17]. Xu [17] reported in grass carp that miR-101a was the most stable miRNA gene in the fish species among the seven miRNAs in their study. In the study, it was confirmed that let-7a, miR-26a and miR-22a were more stable and suitable than miR-101a in Chinese perch. Therefore, they could be used as suitable internal controls for miRNA expression quantification in teleost. However, the geNorm stability value of U6 was 2.23, indicating that U6 is not suitable as internal control in Chinese perch.

In the assays of the embryo samples from different developmental stages, all of the miRNAs were expressed lower than in any other samples. The cycle threshold (*C*_t_) values displayed a wide range from 18.54 to 31.53 (Figure 1B). When data was compared with four algorithms (BestKeeper, geNorm, NormFinder and the comparative Δ-*C*_t_ method), there is an apparent difference among different developmental stages, which suggested that the stability of miRNA expression appears dynamic during embryonic development. However, our results indicate that miR-101a was the most stable reference miRNA in embryonic developmental stages, followed by let-7a, miR-26a, miR-22a, miR-146a, miR-23a and U6 (Figure 2C).

Skeletal muscle is the largest tissue in the fish body and the main edible-part for human consumption. Recent studies have shown that miRNAs play important roles in skeletal muscle development in vertebrates [27,28]. As earlier reported, miR-23a and let-7a were the most stable miRNA in grass carp and pig muscle at different developmental stages [16,17]. In the present study, the *M*-values of miR-23a and let-7a were less than 1.5, indicating that they are also suitable for miRNA expression normalization in fish, at least in Chinese perch muscle.

A crucial criterion for a reference gene is that its expression levels remain constant without bias to treatment, age, tissue type, nutrition, physiological status, *etc*. [29]. Therefore, we conducted fasting-refeeding experiment to investigate the variation of six candidate reference miRNAs. All the *C*_t_ variation ranges of these miRNAs during fasting-refeeding treatment were smaller than those in different tissues and different developmental stages. It was suggested that nutritional factors may have little effect on miRNA expression. Furthermore, based on *M*-values calculated by geNorm, all of the candidate reference miRNAs were suitable for this kind of assay.

A recent study suggested that using multiple reference genes as internal controls is more accurate than using only one single gene for normalization [14]. In the study, by analyses with Normfinder software and geNorm, the results revealed that the best combination of candidate reference genes was miR-22a and miR-23a in all conditions we assayed. The combination of miR-22a and miR-23a is the best for different tissues, miR-22a and miR-146a is the best for embryonic developmental stages, let-7a and miR-26a for post-embryonic developmental stages of muscle, and miR-26a and miR-23a for the liver tissue after nutritional treatment.

## 4. Materials and Methods

### 4.1. Fish and Tissue Sampling

All Chinese perch individuals were reared under standard conditions (22 °C, 12 h of light and dark) at the Xingda fish hatchery, Changde, Hunan, China. Fish tissue samples, including brain, kidney, liver, spleen, heart, intestine, red muscle and white muscle were collected from ten adult individuals (average body weight 500 g, 150 days of post-hatching (dph)). The fast muscle was dissected from each individual fish dorsal myotome of different post-embryonic development stages (20, 30, 50, 70, 90 and 150 (dph), ten individuals from each stage). Samples from different embryonic developmental stages including the embryos at the two-cell stage, blastula stage, gastrula stage, neurula stage, tail-bud stage, muscular effect stage, heart beating stage and larval stage (10 specimens from each stage), were obtained after artificial fertilization until hatching at the Hunan Aquaculture Institute, China. All of the tissues and embryo samples were snap-frozen in liquid nitrogen and stored at −80 °C for further processing.

To investigate expression stability of the miRNAs during fasting and refeeding experiments, 80 Chinese perch juveniles (average body weight 150 g, 90 dph) in a net cage (5 m × 5 m × 2 m) were reared under the same conditions as described above and they were fed with mud carp (average body weight 10 g) for one week. The fish juveniles were fasted for one week, and then fed with a single meal (mud carp). Liver samples were collected at 0 h (before the recovery meal), 1, 3, 6, 12, 24, 48 and 96 h after the single meal feeding respectively (ten individuals from each stage).

### 4.2. RNA Isolation and First Strand cDNA Synthesis

All tissue samples were ground in liquid nitrogen as preprocesses. The total RNAs were extracted from embryos and tissue samples using TRIzol^®^ Reagent (Invitrogen, Waltham, MA, USA) according to the manufacturer’s protocol, and then treated with RNAse-free DNAse I (Promega, Fitchburg, WI, USA) in the presence of RNAse inhibitor (Sigma, Saint Louis, MO, USA). The RNAs were harvested by ethanol precipitation and their concentration and purity were determined by at the absorbance at 260 and 280 nm in a Nanodrop 2000 spectrophotometer (Nanodrop Technologies, Wilmington, DE, USA). The RNA integrity was assessed by the 18S and 28S band intensity ratio after 1.5% agarose gel electrophoresis visualized by GoldView II staining (Beijing, China). Equal amounts of 800 ng/μL of RNAs were polyadenylated by poly (A) polymerase, and then reverse transcribed using the One Step PrimeScript miRNA cDNA synthesis kit (TaKaRa, Dalian, China) with the Universal Adaptor Primer (a poly (T) primer ligated with an adapter) for miRNA quantitative assays.

### 4.3. Quantitative Real-Time PCR Assay

The forward primers for miRNAs RT-qPCR were designed based on the mature miRNA sequence (Table 1), while about 2 to 6 bp was removed at the 3' end and about 2 to 6 bp was added at 5' end according to the Tm value. The primers for U6 RT-qPCR referred to the previous report [30]. Standard curves were generated to calculate the RT-qPCR efficiency. All standard curves were made using 10 fold serial dilutions from a pool of cDNA which consist of cDNA samples from eight tissues (including brain, kidney, liver, spleen, heart, intestine, red muscle and white muscle, *n* = 80). The cDNA samples were used as templates for quantitative RT-PCR assays with SYBR Premix Ex Taq™ II (TaKaRa, Dalian, China) and its amplification reaction was carried out with Bio-Rad CFX96 system (Bio-Rad, Hercules, CA, USA). Each of 2 μL cDNA template was added to a total volume of 25 μL reaction mix containing 12.5 μL SYBR Green mix, 1 μL of miRNA specific forward primer and 1 μL of miRNA universal downstream primer forward primer (Table 1, 10 μmol/L), 8.5 μL nuclease-free water. The following protocol was used: (i) Pre-denaturation at 95 °C for 60 s; (ii) Amplification and quantification, 40 repeated cycles at 95 °C for 5 s and at 60 °C for 25 s; (iii) Melting curve program (65–95 °C with heating rate of 0.1 °C/s and fluorescence measurement).

### 4.4. Selection of Candidate Reference miRNAs and Expression Stability Analyses

The candidate reference miRNAs selected for the evaluation include: miR-101a, miR-146a, miR-22a, miR-23a, miR-26a and let-7a; These candidate miRNAs were previously evaluated as reference miRNAs [16,17,31]. The stability of each candidate miRNA was analyzed with geNorm [14], NormFinder [32], BestKeeper [33] and the comparative Δ-*C*_t_ [34] method algorithm. The geNorm algorithm was used to calculate the gene expression stability (*M*) value based on average pairwise variation between all studied genes. NormFinder was used to analysis of variance (ANOVA) mathematical model and estimates of intra- and inter-group variation. In both programs, the lowest stability values indicate the most stably expressed reference genes, which allow them to be ranked according to expression stability. Based on the rankings from each program, RefFinder (http://www.leonxie.com/referencegene.php) assigns an appropriate value to an individual gene with overall final ranking. Then we ran a validation experiment with comparative profiling of the miR-499 expression in white muscle and red muscle of *Siniperca chuatsi* by microarray analysis and RT-qPCR; For the method of miRNA microarray analysis, please refer to Chu *et al.* [25].

## 5. Conclusions

In the present study we analyzed the suitability of six miRNA genes with four commonly used programs and confirmed that miR-22a was the most suitable single reference gene to investigate gene expression profiles in most of the tested conditions of Chinese perch. The miR-22a and miR-23a was the most suitable combination to normalize miRNA expression in Chinese perch. Our studies also suggested that, the six candidate reference miRNAs were more suitable as reference gene for miRNA quantification than the most commonly used reference gene (U6) in most of the tested conditions. Overall, this study provides valuable information about the Chinese perch reference miRNAs that could be used for gene expression normalization in other teleost species.

## Supplementary Materials

Supplementary materials can be found at http://www.mdpi.com/1422-0067/16/04/8310/s1.
