# Supplementary material for: Selection of Reference Genes for MicroRNA Quantitative Expression Analysis in Chinese Perch, Siniperca chuatsi
Source: Int J Mol Sci. 2015 Apr 14;16(4):8310–23. doi: 10.3390/ijms16048310 (PMC4425082; doi:10.3390/ijms16048310)
Supplement: Supplementary file 1 [file ijms-16-08310-s001.pdf]

Supplementary Information

Part 1. The stability values obtained for each gene by each algorithm in consideration of all samples including different adult tissues, developmental stages and fasting and refeeding experiment.

Table S1–1. Gene stability by Δ-C_t method.

Genes	Average of STDEV
miR-22a	2.34
miR-101a	2.52
let-7a	2.61
miR-23a	2.64
miR-26a	2.65
miR-146a	2.82
U6	6.21

Table S1–2. Crossing point (CP) data of housekeeping Genes by BEST KEEPER.

Factor	let-7a	miR-22a	miR-23a	miR-26a	miR-146a	miR-101a	U6
n	300	300	300	300	300	300	300
geo Mean [CP]	inf	inf	inf	inf	inf	inf	inf
AR Mean [CP]	19.68	20.97	21.03	20.37	22.41	21.86	27.74
Min [CP]	13.37	15.9	15.55	15.09	17.02	16.46	19.67
Max [CP]	33.77	32.2	35.37	34.09	33.38	34.53	36.82
Std. Dev. [± CP]	4.09	3.19	2.41	4.1	3.31	3.69	2.64
CV [% CP]	20.8	15.19	11.44	20.13	14.76	16.89	9.53
Min [*x*-fold]	inf	inf	inf	inf	inf	inf	inf
Max [*x*-fold]	0	0	0	0	0	0	0
Std. Dev. [± *x*-fold]	17.07	9.1	5.3	17.13	9.89	12.93	6.25

inf: Infinitely; N: Number of samples; AR Mean [CP]: The arithmetic mean of CP; Min [CP] and Max [CP]: The extreme values of CP; Std. Dev. [± CP]: The standard deviation of the CP; CV [% CP]: The coefficient of variance expressed as a percentage on the CP level; Min [*x*-fold] and Max [*x*-fold]: The extreme values of expression levels expressed as an absolute *x*-fold over- or under-regulation coefficient; Std. Dev. [± *x*-fold]: Standard deviation of the absolute regulation coefficients.

Table S1–3. Pearson correlation coefficient (*r*) by BEST KEEPER.

	let-7a	miR-22a	miR-23a	miR-26a	miR-146a	miR-101a	U6
miR-22a	0.963	-	-	-	-	-	-
p-Value	0.001	-	-	-	-	-	-
miR-23a	0.873	0.903	-	-	-	-	-
p-Value	-	-	-	-	-	-	-
miR-26a	0.979	0.966	0.864	-	-	-	-
p-Value	-	-	0.001	-	-	-	-
miR-146	0.878	0.857	0.868	0.831	-	-	-
p-Value	-	-	0.001	-	-	-	-
miR-101a	0.962	0.947	0.880	0.963	0.840	-	-
p-Value	0.001	0.001	0.001	0.001	-	-	-
U6	−0.485	−0.433	−0.254	−0.520	−0.272	−0.526	-
p-Value	-	0.001	0.001	0.001	0.001	0.001	-

Table S1–4. Gene stability by normFinder method.

Gene Name	Stability Value
miR-22a	0.342
miR-23a	0.558
miR-146a	1.321
miR-101a	1.445
let-7a	1.837
miR-26a	1.922
U6	6.098

Part 2. The stability values obtained for each gene by each algorithm in consideration of different adult tissues.

Table S2–1. Gene stability by Δ-C_t method.

Genes	Average of STDEV
let-7a	1.72
miR-26a	1.74
miR-22a	1.82
miR-101a	1.90
miR-23a	2.11
miR-146a	2.12
U6	4.19

Table S2–2. Crossing point (CP) data of housekeeping Genes by BEST KEEPER.

Factor	let-7a	miR-22a	miR-23a	miR-26a	miR-146a	miR-101a	U6
n	80	80	80	80	80	80	80
geo Mean [CP]	17.09	18.96	19.05	18.08	20.86	18.64	29.88
AR Mean [CP]	17.37	19.18	19.35	18.35	21.07	18.91	29.96
Min [CP]	14.35	15.9	15.55	15.49	17.39	16.46	25.19
Max [CP]	33.77	32.2	35.37	34.09	33.38	34.53	35.93
Std. Dev. [± CP]	1.98	1.96	2.24	1.96	2.18	2.15	1.66
CV [% CP]	11.41	10.2	11.56	10.67	10.36	11.37	5.55
Min [*x*-fold]	−6.68	−8.36	−11.31	−6.03	−11.05	−4.53	−25.89
Max [*x*-fold]	104,770.74	9652.25	81,332.6	65,731.28	5870.55	60,769.65	66.06
Std. Dev. [± *x*-fold]	3.95	3.88	4.71	3.89	4.54	4.44	3.17

n: Number of samples; AR Mean [CP]: The arithmetic mean of CP; Min [CP] and Max [CP]: The extreme values of CP; Std. Dev. [± CP]: The standard deviation of the CP; CV [% CP]: The coefficient of variance expressed as a percentage on the CP level; Min [*x*-fold] and Max [*x*-fold]: The extreme values of expression levels expressed as an absolute *x*-fold over- or under-regulation coefficient; Std. Dev. [± *x*-fold]: Standard deviation of the absolute regulation coefficients.

Table S2–3. Pearson correlation coefficient (*r*) by BEST KEEPER.

	let-7a	miR-22a	miR-23a	miR-26a	miR-146a	miR-101a	U6
miR-22a	0.973	-	-	-	-	-	-
p-Value	-	-	-	-	-	-	-
miR-23a	0.915	0.889	-	-	-	-	-
p-Value	-	-	-	-	-	-	-
miR-26a	0.982	0.970	0.938	-	-	-	-
p-Value	-	-	-	-	-	-	-
miR-146	0.875	0.858	0.884	0.869	-	-	-
p-Value	0.001	-	0.001	0.001	-	-	-
miR-101a	0.960	0.918	0.913	0.939	0.919	-	-
p-Value	0.001	0.001	-	-	-	-	-
U6	0.064	0.006	0.040	0.048	0.020	-0.001	-
p-Value	0.629	0.964	0.760	0.716	0.879	0.994	-

Table S2–4. Gene stability by normFinder method.

Gene Name	Stability Value
let-7a	0.758
miR-22a	0.771
miR-26a	0.822
miR-101a	1.112
miR-146a	1.251
miR-23a	1.423
U6	4.059

Part 3. The stability values obtained for each gene by each algorithm in consideration of different embryonic developmental stages.

Table S3–1. Gene stability by Δ-C_t method.

Genes	Average of STDEV
miR-22a	1.11
let-7a	1.12
miR-26a	1.17
miR-101a	1.20
miR-146a	1.30
miR-23a	1.34
U6	2.33

Table S3–2. Crossing point (CP) data of housekeeping Genes by BEST KEEPER.

Factor	let-7a	miR-22a	miR-23a	miR-26a	miR-146a	miR-101a	U6
n	80	80	80	80	80	80	80
geo Mean [CP]	25.88	25.55	23.72	26.74	25.49	27.39	24.01
AR Mean [CP]	25.97	25.68	23.9	26.82	25.63	27.45	24.11
Min [CP]	21.44	20.21	18.55	23.14	20.13	24.37	19.67
Max [CP]	30.02	30.26	29.18	30.65	30	31.53	29.76
Std. Dev. [± CP]	1.68	2.02	2.37	1.68	2.28	1.53	1.75
CV [% CP]	6.46	7.87	9.9	6.25	8.88	5.57	7.25
Min [*x*-fold]	−21.8	−40.49	−36.09	−12.12	−41.16	−8.12	−20.25
Max [*x*-fold]	17.59	26.14	44.13	14.98	22.77	17.62	53.82
Std. Dev. [± *x*-fold]	3.2	4.06	5.15	3.2	4.85	2.89	3.36

n: Number of samples; AR Mean [CP]: The arithmetic mean of CP; Min [CP] and Max [CP]: The extreme values of CP; Std. Dev. [± CP]: The standard deviation of the CP; CV [% CP]: The coefficient of variance expressed as a percentage on the CP level; Min [*x*-fold] and Max [*x*-fold]: The extreme values of expression levels expressed as an absolute *x*-fold over- or under-regulation coefficient; Std. Dev. [± *x*-fold]: Standard deviation of the absolute regulation coefficients.

Table S3–3. Pearson correlation coefficient (*r*) by BEST KEEPER.

	let-7a	miR-22a	miR-23a	miR-26a	miR-146a	miR-101a	U6
miR-22a	0.951	-	-	-	-	-	-
p-Value	0.001	-	-	-	-	-	-
miR-23a	0.952	0.967	-	-	-	-	-
p-Value	-	-	-	-	-	-	-
miR-26a	0.916	0.952	0.951	-	-	-	-
p-Value	-	-	-	-	-	-	-
miR-146	0.926	0.968	0.943	0.931	-	-	-
p-Value	0.001	-	-	-	-	-	-
miR-101a	0.925	0.936	0.933	0.92	0.908	-	-
p-Value	0.001	-	-	-	0.001	-	-
U6	0.566	0.485	0.526	0.439	0.395	0.573	-
p-Value	0.001	0.001	0.001	0.001	0.001	0.001	-

Table S3–4. Gene stability by normFinder method.

Gene Name	Stability Value
let-7a	0.351
miR-101a	0.559
miR-22a	0.572
miR-26a	0.600
miR-23a	0.973
miR-146a	0.975
U6	2.238

Part 4. The stability values obtained for each gene by each algorithm in consideration of different post-embryonic developmental stages.

Table S4–1. Gene stability by Δ-C_t method.

Genes	Average of Stdev
miR-23a	1.02
miR-26a	1.08
miR-22a	1.12
let-7a	1.14
miR-101a	1.16
miR-146a	1.16
U6	2.96

Table S4–2. Crossing point (CP) of housekeeping Genes by BEST KEEPER.

Factor	let-7a	miR-22a	miR-23a	miR-26a	miR-146a	miR-101a	U6
n	60	60	60	60	60	60	60
geo Mean [CP]	19.65	20.47	21.43	19.23	24.9	21.51	29.06
AR Mean [CP]	19.73	20.56	21.51	19.32	24.96	21.6	29.16
Min [CP]	17.39	18.71	19.57	17.51	22.69	19.25	23.95
Max [CP]	25.59	28.49	28.25	25.6	30.02	28.01	36.82
Std. Dev. [± CP]	1.31	1.4	1.32	1.39	1.2	1.48	1.77
CV [% CP]	6.61	6.82	6.14	7.21	4.79	6.86	6.09
Min [*x*-fold]	−4.82	−3.39	−3.62	−3.31	−4.64	−4.78	−34.49
Max [*x*-fold]	61.36	258.77	113.13	82.48	34.78	90.65	217.07
Std. Dev. [± *x*-fold]	2.47	2.64	2.5	2.63	2.29	2.79	3.42

n: Number of samples; AR Mean [CP]: The arithmetic mean of CP; Min [CP] and Max [CP]: The extreme values of CP; Std. Dev. [± CP]: The standard deviation of the CP; CV [% CP]: The coefficient of variance expressed as a percentage on the CP level; Min [*x*-fold] and Max [*x*-fold]: The extreme values of expression levels expressed as an absolute *x*-fold over- or under-regulation coefficient; Std. Dev. [± *x*-fold]: Standard deviation of the absolute regulation coefficients.

Table S4–3. Pearson correlation coefficient (*r*) by BEST KEEPER.

	let-7a	miR-22a	miR-23a	miR-26a	miR-146a	miR-101a	U6
miR-22a	0.924	-	-	-	-	-	-
p-Value	0.001	-	-	-	-	-	-
miR-23a	0.958	0.949	-	-	-	-	-
p-Value	-	-	-	-	-	-	-
miR-26a	0.894	0.949	0.963	-	-	-	-
p-Value	-	0.001	-	-	-	-	-
miR-146	0.934	0.921	0.924	0.911	-	-	-
p-Value	-	-	-	-	-	-	-
miR-101a	0.928	0.939	0.954	0.946	0.907	-	-
p-Value	-	-	-	0.001	0.001	-	-
U6	0.044	0.140	0.188	0.212	0.105	0.122	-
p-Value	0.750	0.314	0.173	0.124	0.450	0.380	-

Table S4–4. Gene stability by normFinder method.

Gene Name	Stability Value
miR-23a	0.249
miR-26a	0.346
miR-146a	0.531
miR-22a	0.588
let-7a	0.638
miR-101a	0.688
U6	2.911

Part 5. The stability values obtained for each gene by each algorithm in consideration of fasting and refeeding experiment.

Table S5–1. Gene stability by Delta C_t method.

Genes	Average of STDEV
miR-23a	0.93
miR-26a	0.97
miR-146a	0.98
let-7a	1.00
miR-22a	1.24
miR-101a	1.28
U6	2.13

Table S5–2. Crossing point (CP) data of housekeeping Genes by BEST KEEPER.

Factor	let-7a	miR-22a	miR-23a	miR-26a	miR-146a	miR-101a	U6
n	80	80	80	80	80	80	80
geo Mean [CP]	15.23	18.04	19.19	16.36	18.37	18.91	28.4
AR Mean [CP]	15.25	18.06	19.21	16.37	18.38	18.93	28.47
Min [CP]	13.37	16.31	17.48	15.09	17.02	17.4	24.73
Max [CP]	17.16	19.86	20.64	17.77	20.5	21.03	32.34
Std. Dev. [± CP]	0.73	0.8	0.69	0.62	0.6	0.72	1.67
CV [% CP]	4.81	4.42	3.62	3.77	3.24	3.81	5.86
Min [*x*-fold]	−3.62	−3.32	−3.28	−2.41	−2.54	−2.85	−12.75
Max [*x*-fold]	3.82	3.53	2.73	2.66	4.39	4.34	15.32
Std. Dev. [± *x*-fold]	1.66	1.74	1.62	1.53	1.51	1.65	3.18

n: Number of samples; AR Mean [CP]: The arithmetic mean of CP; Min [CP] and Max [CP]: The extreme values of CP; Std. Dev. [± CP]: The standard deviation of the CP; CV [% CP]: The coefficient of variance expressed as a percentage on the CP level; Min [*x*-fold] and Max [*x*-fold]: The extreme values of expression levels expressed as an absolute *x*-fold over- or under-regulation coefficient; Std. Dev. [± *x*-fold]: Standard deviation of the absolute regulation coefficients.

Table S5–3. Pearson correlation coefficient (*r*) by BEST KEEPER.

	let-7a	miR-22a	miR-23a	miR-26a	miR-146	miR-101a	U6
miR-22a	0.321	-	-	-	-	-	-
p-Value	0.006	-	-	-	-	-	-
miR-23a	0.905	0.34	-	-	-	-	-
p-Value	-	0.004	-	-	-	-	-
miR-26a	0.606	0.304	0.716	-	-	-	-
p-Value	0.001	0.009	0.001	-	-	-	-
miR-146a	0.661	0.147	0.768	0.809	-	-	-
p-Value	0.001	0.217	0.001	-	-	-	-
miR-101a	0.142	−0.034	0.162	0.464	0.44	-	-
p-Value	0.233	0.775	0.175	0.001	0.001	-	-
U6	0.219	0.331	0.251	−0.124	−0.135	−0.339	-
p-Value	0.064	0.004	0.034	0.3	0.258	0.004	-

Table S5–4. Gene stability by normFinder method.

Gene Name	Stability Value
miR-23a	0.245
let-7a	0.451
miR-26a	0.477
miR-146a	0.519
miR-22a	0.813
miR-101a	1.002
U6	2.039
